# Genetic and Statistical Study of Anelloviruses and Gyroviruses in Diarrheic Cats and Their Co-Occurrence Patterns

**DOI:** 10.3390/v17111413

**Published:** 2025-10-23

**Authors:** Turhan Turan, Hakan Işıdan, Selda Duran-Yelken, Mustafa Ozan Atasoy, Remziye Özbek, Rania F. El Naggar, Mohammed A. Rohaim

**Affiliations:** 1 Department of Veterinary Virology, Faculty of Veterinary Medicine, Cumhuriyet University, 58140 Sivas, Türkiye; tturan@cumhuriyet.edu.tr (T.T.); hisidan@cumhuriyet.edu.tr (H.I.); mozan@cumhuriyet.edu.tr (M.O.A.); remziyeozbek@cumhuriyet.edu.tr (R.Ö.); 2 Department of Veterinary Virology, Faculty of Veterinary Medicine, Kastamonu University, 37150 Kastamonu, Türkiye; syelken@kastamonu.edu.tr; 3 Department of Virology, Faculty of Veterinary Medicine, University of Sadat City, Sadat 32897, Egypt; rania.elnagar@vet.usc.edu.eg; 4 Department of Virology, Faculty of Veterinary Medicine, Cairo University, Giza 12211, Egypt

**Keywords:** feline, anelloviruses, gyroviruses, parvoviruses, feline panleukopenia virus, phylogeny, sequencing

## Abstract

Members of the *Anelloviridae* family are increasingly being recognized for their role in veterinary and public health, with domestic cats identified as potential carriers of anelloviruses and gyroviruses. This study aimed to investigate the prevalence and genetic characteristics of these viruses in diarrheic cats from Sivas, Türkiye. A total of 91 fecal samples were analysed, initially for feline panleukopenia virus using conventional PCR, followed by screening with our *Anelloviridae* panel. The results revealed that 19 (20.9%) samples were positive for TTFeV1, 32 (35.2%) for CAV, 67 (73.6%) for Avian gyrovirus 2, four (4.4%) for Gyrovirus 3, and three (3.3%) for Gyrovirus 4. Statistical analyses revealed frequent co-infections among parvoviruses, anelloviruses, and gyroviruses, with a significant association between *Gyrovirus chickenanemia* (CAV) and *Gyrovirus galga1* (AvGyV2). Notably, Gyrovirus 4 (*Gyrovirus homsa3*) was identified in feline stool for the first time. Phylogenetic and genomic analyses, based on partial TATA box-ORF2 sequences for anelloviruses and VP1 sequences for gyroviruses, provided further insights into viral diversity. These findings expand current knowledge of anellovirus and gyrovirus circulation in feline populations, underscoring the importance of continued surveillance for feline and public health.

## 1. Introduction

The family *Anelloviridae* contains negative-sense, circular, and single-stranded DNA viruses with a genome of approximately 1.6–3.9 kb in length [1]. Anelloviruses were first described in Japan in 1997 in a patient who developed hepatitis as a result of blood transfusion [2]. Since then, they have been detected in many animal species such as pigs, non-human primates, marine mammals, bats, dogs, cats, ruminants, and equids [3,4,5,6,7]. Additionally, in cervical specimens, TTVs have been discovered to co-infect with other viruses, such as human papillomavirus, indicating the possibility of interactions between several viral species [8]. The *Anelloviridae* family includes 30 genera with high genetic heterogenity and 156 species belonging to these genera. According to recent update on the International Committee on Taxonomy of Viruses (ICTV), feline anelloviruses have been classified under the in the genus *Etatorquevirus* (*Etatorquevirus felid1–5*) and *Tettorquevirus* (*Tettorquevirus felid6*). In addition to these two genera, four of the 10 species in *Gyrovirus* genus, Chicken anemia virus (CAV), also known as *Gyrovirus chickenanemia*; *Gyrovirus galga1* (previously referred to as *Avian gyrovirus 2*, or AvGyV2); *Gyrovirus 3* (GyV3), also called “*Gyrovirus homsa1*”; and *Gyrovirus 6* (GyV6) have been identified in cats so far [9,10,11,12,13].

Anelloviruses are highly prevalent in human and animal populations, but their importance on health remains uncertain. Myriads of studies have been conducted on Torque teno viruses (TTVs), pinpointing their significance as novel agents that could affect the health of humans and other animals. After the first report of feline TT viruses by Okamoto et al. (2002), the CAV genome was detected in breeder and commercial chicken flocks in South Korea [5,14]. A year later, a new human virus was discovered on the human skin and named “human gyrovirus” (HGyV) due to its similarity to the chicken anemia virus [15]. In the same year, another species of gyrovirus related to CAV, known as *Avian gyrovirus 2* (AvGyV2), was identified in diseased chickens in Brazil [16]. Due to the high nucleotide identity of over 93% between HGyV and AvGyV2 in the VP1-VP3 gene, they have been regarded as the same species and given the name HGyV/AGV2. The genus Gyrovirus formally includes eleven species that have been discovered in a wide range of hosts, including chickens, birds, ferrets, humans, dogs, and cats [17,18]. Chicken anemia virus is a highly pathogenic and contagious viral agent characterized by severe aplastic anaemia, subcutaneous and muscular haemorrhages, thymus atrophy, abnormal feather development, and profound immunosuppression, thereby predisposing affected young chickens to secondary infections caused by various pathogens [19]. The GyV3 was detected in both diarrheal and formed stool samples from Chilean children in the USA, while *Gyrovirus 4* (GyV4), or “*Gyrovirus homsa3*” has been detected in human and chicken stool, also raw chicken [20,21].

While well-known viruses like *protoparvovirus carnivoran1*, which is an aetiological agent of feline panleukopenia (otherwise known as feline distemper), have been extensively studied due to their major impact on domestic cats, the emergence of new viruses and their potential role in gastroenteric diseases often remain unforeseen. To address this gap, we aimed to investigate the prevalence of feline torque teno viruses, specifically Torque teno felid virus 1 (TTFeV1) and 2 (TTFeV2), as well as four different Gyrovirus species, in the diarrheal fecal samples of domestic cats, providing insight into their possible contribution to feline gastrointestinal disorders.

## 2. Materials and Methods

### 2.1. Sampling

A total of 91 rectal swab samples were collected between May 2019–May 2021 from cats suffering from mild to severe diarrhea. Samples were obtained from animals which was admitted to Sivas Cumhuriyet University Faculty of Veterinary Medicine, and six other private district veterinary clinics located in the Sivas province. Of the 91 rectal swab samples, 56 were from kittens under one year old, and 35 were from adult cats aged 1 to 8 years. The collected samples were transported to the laboratory just after sampling and stored at −80 °C until being subjected to DNA isolation. Samples were diluted to 1:10 with 1 M phosphate-buffered saline solution and centrifuged at 3000× *g* for 5 min to remove coarse particles. After centrifugation, the supernatants were submitted to a nucleic acid extraction procedure using a GF-1 Viral Nucleic Acid Extraction Kit (Vivantis, Subang Jaya, Malaysia) according to the manufacturer’s instructions. The eluted DNAs were stored at −80 °C until use.

### 2.2. Polymerase Chain Reactions (PCRs)

DNA samples were initially screened by conventional PCR targeting the VP2 gene of *Protoparvovirus carnivoran1*, also known as Feline panleukopenia virus (FPV) in feline species using primer set previously described by Buonavoglia et al. (2001) [22]. The same samples were further investigated to detect feline anelloviruses and gyroviruses. All primers used in this study are listed in Table 1. All Reactions were performed in 30 µL volume using a commercial PCR master mix (Thermo, Waltham, MA, USA). PCR conditions were adjusted as follows: Following pre-denaturation at 95 °C for 2 min., 45 s at 94 °C for denaturation, annealing at 48–57 °C (listed in Table 1) for 30 s and 1 min. at 72 °C for extension were set to 40 cycles. Final extension was at 72 °C for 10 min. Amplified products were run on the 1% agarose gel containing GelRed Nucleic Acid Stain (Merck, Rahway, NJ, USA) and visualised by a standard transilluminator. The samples showing the expected band size were regarded as positive.

### 2.3. Sequencing and Phylogenetic Analysis

Sanger sequencing and BLAST (v.2.16.0) comparison confirmed that the PCR product was the predicted gene region length (Table 1). PCR amplicons were purified using Wizard SV Gel and PCR CleanUp System (Promega, Madison, WI, USA) and BigDye Terminator v1.1 Cycle Sequencing Kit (Applied Biosystems, Foster City, CA, USA) and ABI Prism 310 Genetic Analyzer (ABI 3100; Applied Biosystems, Foster City, CA, USA). Among the positive samples, six Torque teno felis virus 1, five CAV, five AvGyV2, four Gyv3 and three GyV4 samples were randomly selected for bidirectional dideoxy sequencing. Partial sequences of six TATA Box-ORF2 gene of TTFeV1 were compared with other available strains retrieved from GenBank. Phylogenetic analysis of partial VP1 gene sequences of gyroviruses (CAV, AvGyV2, GyV3, and GyV4) were performed and compared with other publicly available anellovirus data in the NCBI database.

The phylogenetic analysis was conducted using MEGAX (v.10.2.6) software by applying the maximum likelihood (ML) method [23]. MEGAX software was utilized to evaluate the Bayesian Information Criterion (BIC) in order to select the best-fit substitution model. The model with the lowest BIC value was chosen as it represents the best fit for the data. Then phylogenetic trees were generated using the maximum likelihood method and the Kimura-2 parameter model (+G) with 1000 replicates [24]. The nucleotide (nt) identities were calculated using the SIAS on-line tool (http://imed.med.ucm.es/Tools/sias.html; accessed on 2 November 2024). The datasets were individually analysed for potential recombination events in both structural and non-structural genes using RDP4 v4.100 software, applying multiple algorithms (RDP, GENECONV, Chimaera, Max-Chi, BootScan, SiScan, and 3Seq) with default parameters and a *p*-value threshold of 0.05 [25].

### 2.4. Statistical Analyses

All statistical analyses and visualizations were performed using RStudio (version 2024.09.0) (http://www.rstudio.com/). Initially, the statistical relationship between feline panleukopenia virus (FPV) and the other viruses subjected to this study (circoviruses and anelloviruses) was examined individually using a chi-squared test, along with calculations of both phi coefficient and Cramér’s V to assess the direction and strength of the associations. Then, pairwise comparisons were performed among the remaining viruses using the same methodology to identify any significant associations.

## 3. Results

### 3.1. Prevalence of Feline Panleukopenia Virus

**Figure 1:**
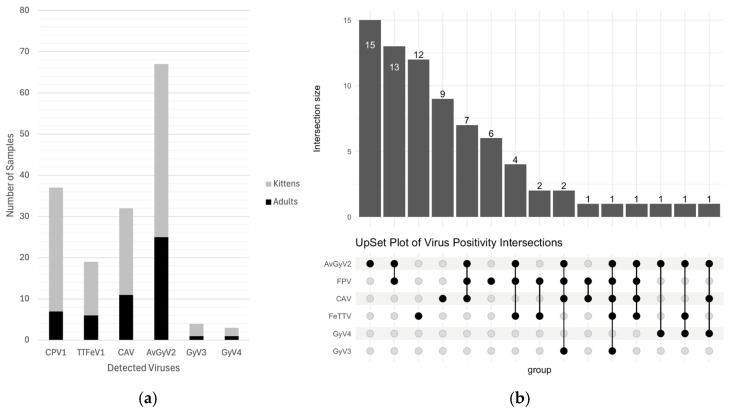
(**a**) Graphical Representation of PCR Assay-Positive Samples A. Distribution of positive samples by age. Cats were categorized into two groups based on age: kittens (<1 year old) and adults (1–8 years old); (**b**) UpSet plot showing co-occurrence patterns of six viruses. Each column in the plot represents a unique combination of virus positivity among FPV, AvGyV2, FeTTV, CAV, GyV3, and GyV4. Filled dots in the lower matrix indicate which viruses are present in each combination, and vertical bars above show the number of samples with that specific co-occurrence.

From the 91 samples initially investigated, 37 samples were positive for feline panleukopenia virus (40.65%). The positivity rate varied significantly across age groups: only 20% (7/35) of the adult cats tested positive for FPV, while the rate peaked at 53.57% (30/56) among kittens (Figure 1a). PCR detection results for the seven viruses in adults and kittens are summarized in Table 2.

### 3.2. Prevalence of the Anelloviridae Family

PCR positivity for anelloviruses and gyroviruses was detected in 75 of 91 rectal swab samples of cats. Of the 56 kitten samples, 13 (23.21%) were positive for TTFeV1, 21 (37.50%) for CAV, 42 (75.00%) for AvGyV2, 3 (5.36%) for GyV3, and 2 (3.57%) for GyV4, whereas, of the 35 adult cat samples, 6 (17.14%) were positive for TTFeV1, 11 (31.43%) for CAV, 25 (71.43%) for AvGyV2, 1 (3.86%) for GyV3, and 1 (3.86%) for GyV4 (Figure 1a). Furthermore, a single anellovirus infection was detected in 23 cases among the 56 kittens with diarrhea. Mixed infections were observed in the remaining kittens: 15 had dual infections, eight had triple infections, and one had a quintuple infection. Furthermore, among the 35 adult cats with diarrhea, 13 had a single infection, while mixed infections were detected in the others: 14 had dual infections, and only one had a triunal infection (Figure 1b).

### 3.3. Genetic Profiling and Characteristics of Anelloviruses and Gyroviruses

After PCRs, six samples for TTFeV1, five samples for CAV, five samples for AvGyV2, four samples for GyV3, and three samples for GyV4 were successfully sequenced for molecular analysis.

#### 3.3.1. Molecular Analysis of TTFeV1

**Figure 2:**
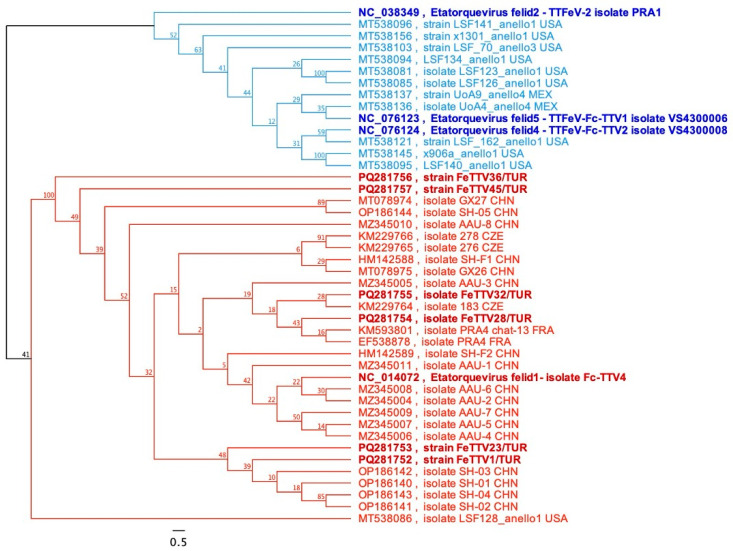
Phylogenetic tree based on the partial sequences of the six TATA Box-ORF2 genes of TTFeV1 detected in this study, along with other related strains retrieved from GenBank. Reference sequences and isolates found in this study are shown in bold. The K80 substitution model was used to generate the tree, with 100 bootstrap replicates.

A phylogenetic tree was constructed with 330 bp-partial sequences of the six TATA Box-ORF2 genes of TTFeVs in this study retrieved from GenBank. Phylogenetic analysis revealed a distinctive distribution between the TTFeV1 and TTFeV2 genera, which were supported by a high bootstrap value (100%). All Turkish strains clustered together with the strain Fc-TTV4 (NC_014072), and some other strains majorly reported from China (Figure 2). The identity percentage of TTFeV1 viruses ranged between 96.36% and 99.39% among themselves. Furthermore, five of the six TTFeV1 samples were more closely related to Chinese strains with a range of identity, while sample FeTTV28 was identical with PRA4 and chat-13 FRA (KM593801) and (EF538878) isolates previously reported from cat saliva (Figure 2).

#### 3.3.2. Molecular Analysis of Chicken Anemia Virus

**Figure 3:**
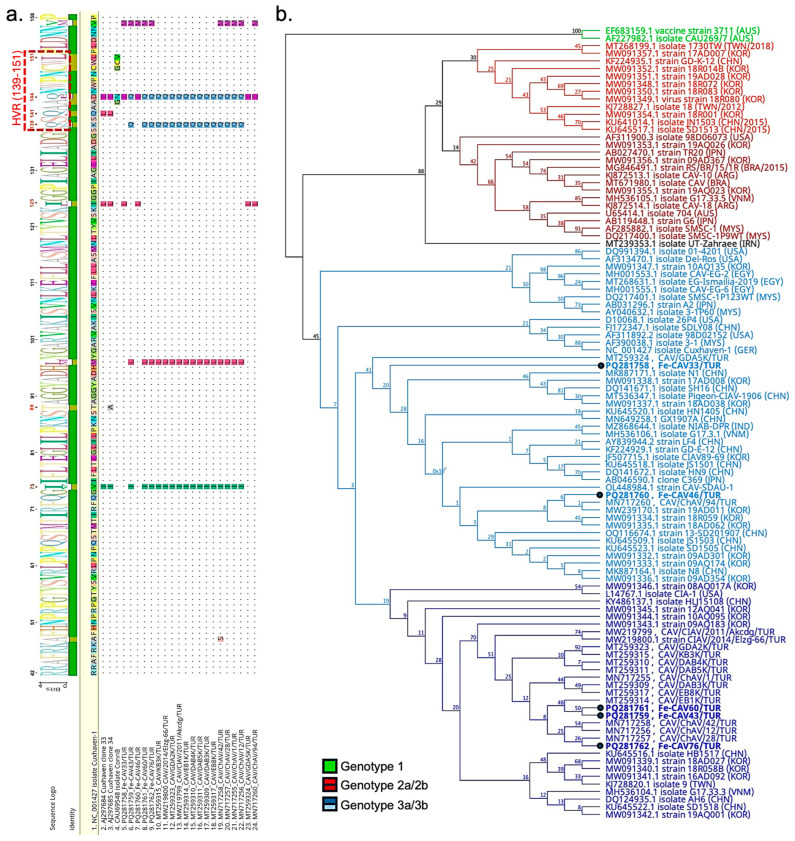
(**a**) Phylogenetic tree based on the partial sequences of VP2 gene of Chicken Anemia Virus (CAV) detected in this study along with other related strains retrieved from GenBank. The PYML tree was made using JC69 substitution model and bootstrapped 100 times. The dark and light colours of each clade represent the subgroups of the respective groups. (**b**) Multiple sequence analysis was performed based on the 117 amino acid sequence of the VP1 gene. The figure was generated using all Turkish strains, the reference strain, and attenuated strains. The hypervariable domain is highlighted in a box, and critical mutations are marked in red numbers.

Pairwise sequence analysis of 351 bases revealed that the five strains were closely related, with high identity ranging from 96.30% to 100%. Notably, Fe-CAV43/TUR and Fe-CAV60/TUR were identical based on partial sequences. However, overall identity dropped to 94.02% when additional Turkish strains were included. Further analysis using the reference strain Cuxhaven-1 (NC_001427.1) identified various potential polymorphisms in the 17 deduced amino acid sequences of the Cux-1 gene and the 117 amino acid sequences of the nucleocapsid gene (ORF3). These mutations were compared with other partial sequences, including strains previously reported from Türkiye. The partial alignment results revealed point mutations at positions V75, M97, I125, K139, D144, and V157 in the capsid protein sequence (Figure 3a). Notably, these mutations were also observed in other Turkish strains. Furthermore, the C-terminal end of the Cux-1 protein was identical to the reference sequence. However, non-synonymous mutations in Fe-CAV43/TUR and Fe-CAV60/TUR resulted in a conversion from opal (TGA) to ochre (TAA), a change also observed in the isolate EB1K, which was previously identified from a broiler.

Phylogenetic analysis using 351-base nucleotide sequences classified the samples into three distinct groups (Genotypes I–III) with strong bootstrap values. Our strains primarily clustered within Group III, further branching into multiple subgroups. Fe-CAV43/TUR, Fe-CAV60/TUR, and Fe-CAV76/TUR formed a distinct branch alongside other strains previously reported from Türkiye, while Fe-CAV33/TUR and Fe-CAV46/TUR diverged from this main branch (Figure 3b).

#### 3.3.3. Molecular Analyses of *Avian gyrovirus 2*, *Avian gyrovirus 3* and *Avian gyrovirus 4*

Despite being classified under the Anelloviridae genus, multiple sequence analyses of partial nucleocapsid sequences for GyV2, GyV3, and GyV4 were applied individually due to high dissimilarity within the species. The 609 bp nucleotide and 203 derived amino acid (aa) sequences of the partial VP1 gene of AvGyV2 were utilised to conduct multiple sequence alignments with reference sequence (Clone Ave3, NC_015396.1). Turkish strains had varied nucleotide identity between 92.78% and 98.36%. FeAvGyV2-31/TUR showed high identity to reference genome (99.51%), whereas FeAvGyV2-46 was identical to two Chinese strains, isolate HLJ1508 (KX708510) and strain 27-GD201810 (OQ116651), both isolated from chickens. Other two strains, FeAvGyV2-38/TUR and FeAvGyV2-49/TUR, possessed varied degree of identity to other strains dominantly isolated from China. On the other hand, FeAvGyV2-20/TUR showed slight deviation from other Turkish strains, exhibiting 98.52% identity to the isolate JP/KGSM/M0313-2Li/97, which was obtained from cryopreserved organs of a broiler in Japan in 1997. Furthermore, the deduced amino acid sequences of partial AvGyV2 VP1 sequences revealed a series of substitutions compared to the reference sequence, S154A substitution in three samples (FeAvGyV2-20, 38, 49), R209S in FeAvGyV2-49, R212K and G242R in five samples (FeAvGyV2-20,-31,-38,-46,-49), A270S in four samples (FeAvGyV2-20,-38,-46,-49), V288Q and G293Q in three samples (FeAvGyV2-38,-46,-49), and Q310E in three samples (FeAvGyV2-20,-46,-49). In addition, the V288I mutation in FeAvGyV2-20 was found to be unique.

**Figure 4:**
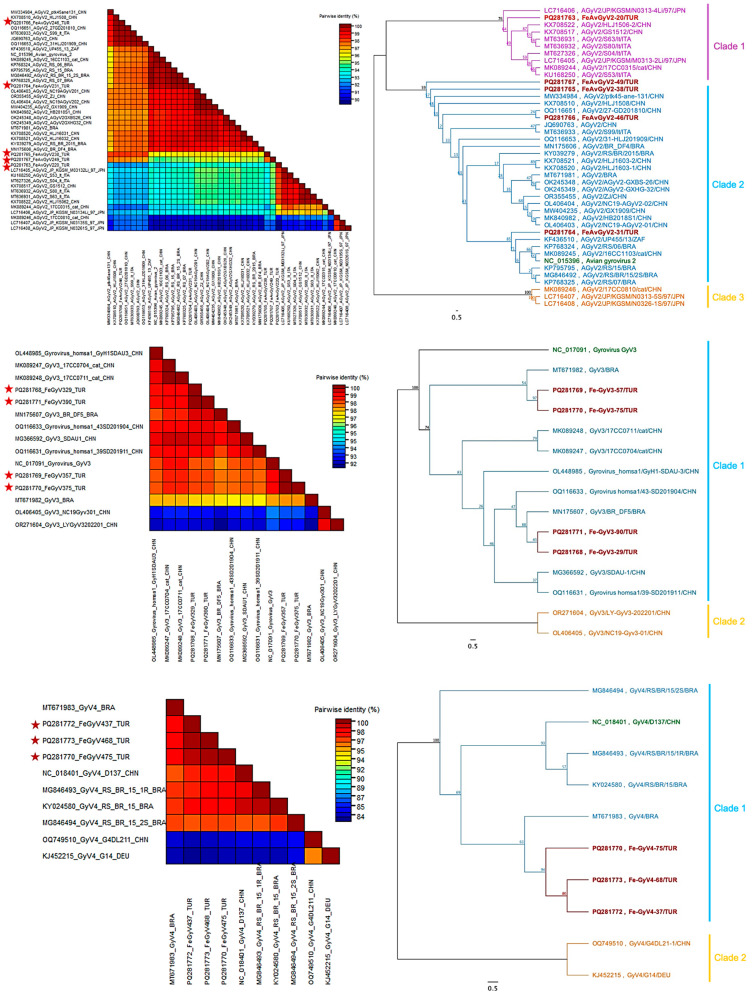
Sequence demarcation analyses of AvGyV2, GyV3, and GyV4 (arranged from top to bottom) are shown on the left, while the corresponding PHYML phylogenetic trees are displayed on the right. Strains in the trees are represented by different colours: the reference strain is shown in green, and Turkish strains are highlighted in bold red. The sequence demarcation graphs further support the phylogenetic results, with Turkish strains marked by red stars. The PHYML trees and sequence demarcation graphs of GyV3 and GyV4 were generated using the same approach.

Phylogenetic trees were built using 609 bp partial VP1 nucleotide sequences of other available strains in GenBank (*n* = 42). Phylogenetic analysis of AvGyV2 revealed two main clades, supported by a high bootstrap value (100%), with Turkish strains clustering in clade 1 as displayed in Figure 4 (top). Clade 1 was further divided into subclades 1a and 1b, supported by a significant bootstrap value (76%). FeAvGyV2-20/TUR was grouped into subclade 1a, while the remaining Turkish strains clustered in subclade 1b along with the reference genome.

The 642 bp nucleotide sequence and the 214 amino acid (aa) sequence derived from the partial VP1 gene of GyV3 were used to perform multiple sequence alignments with the reference sequence (strain FecGy, NC_017091). The Turkish strains exhibited a low level of nucleotide variation, ranging from 98.60% to 100%, with Fe-GyV3-29/TUR and Fe-GyV3-90/TUR being identical to each other. These two strains were closely related (99.53%) to isolate BR_DF5 (MN175607.1), identified from wild bird feces in Brazil. In contrast, Fe-GyV3-57/TUR and Fe-GyV3-75/TUR sequences showed the highest nucleotide identities (98.75%) with two GyV3 strains from China, which were isolated from domestic cat feces (isolate 17CC0704, MK089247; isolate 17CC0711, MK089248.1). The deduced amino acid sequences of Turkish strains were compared with the reference sequence (NC_017091) and all of them found to be identical. A phylogenetic tree was further constructed based on 15 available strains from GenBank, revealing two main clades as displayed in Figure 4 (middle). Clade 1 included the reference sequence and the majority of strains, including Turkish strains, with a minimum identity of 97.35%. Clade 2 comprised only two strains (GyV3/LY-GyV3-202201/CHN, OR271604 and GyV3/NC19-Gyv3-01/CHN, OR271605), sharing 92.52% to 94.24% identity with Clade 1.

The 678 bp nucleotide sequence and the 226 amino acid (aa) sequence derived from the partial VP1 gene of GyV4 were used to perform multiple sequence alignments with the reference sequence (strain GyV4/D137/CHN, NC_018401) along with six other available strains. Our three Turkish strains showed high identity, ranging from 99.41% to 99.71%, and exhibited a close percentage of identity (98.08–98.38%) with a Brazilian strain (GyV4/BRA, MT671983) isolated from chicken meat. Furthermore, the 226-deduced amino acid sequence of Turkish strains displayed various point mutations: the Y116H substitution was detected in Fe-GyV4-37/TUR and found to be unique, whereas the S153T, S175A, and L244T substitutions were detected in all three samples. Additionally, the phylogenetic tree revealed two main clades, Clade 1 and Clade 2, with the Turkish strains falling into Clade 1 as shown in Figure 4 (bottom).

## 4. Discussion

Diarrhea in kittens is frequently considered as one of the significant pre-mortem risk factors due to its high incidence, leading to malabsorption, weight loss, cachexia, and death in the early weeks of life [26]. Kitten diarrhea can be attributed to wide range of factors, including bacterial and viral diseases, such as colibacillosis [27], or parvoviruses [28], drug intoxication [29], or food sensitivity [30]. To better understand the nature of viral diarrhea in kittens and implement effective mitigation measures, it is important to address other pathogens that may serve as contributing factors. Therefore, we aimed to investigate the existence and prevalence of various anelloviruses and gyroviruses in fecal samples from domestic cats exhibiting diarrhea. Our samples were initially investigated using a universal primer set detecting feline panleukopenia virus. Our results revealed an overall FPV positivity rate of 40.65% (37/91) in the investigated samples. The prevalence was notably higher in kittens at 53.57% (30/56) but declined to 20% (7/35) in adult cats. Several endeavours have been made in various provinces of Türkiye, all of which resulted in an overall prevalence between 10% and 25%, varying based on factors such as sample size, location, methodology, cats’ age, and habitat [31,32]. Similarly, many reports have been documented worldwide, including in China, with a prevalence ranging from 8.54% to 45.09% [33,34], 11.32% in India [35], 22.90% in Bangladesh [36], and 43.00% in Egypt [37]. Furthermore, young kittens are known to be more susceptible to infection than adult cats over two years old [38,39]. On the other hand, ten samples (10.99%) yielded negative results for all viruses investigated in this study, while among the 37 FPV-positive samples, six were positive only for FPV (6.59% in total) with no other viruses detected through our *Anelloviridae* panel. FPV may occur alone or in conjunction with other viruses that share similar pathogenic mechanisms, such as astroviruses, caliciviruses, kobuviruses, and other parvovirus species, which can either cause independent infections or contribute to concurrent infections [40,41,42]. Overall, our findings demonstrated a relatively high prevalence of FPV in the Sivas Region, particularly in kittens, which was somewhat consistent with previous reports. Nevertheless, the contribution of other major diarrheal viruses to the clinical manifestations of FPV infection remains to be elucidated.

Torque teno felis viruses, first identified in Japan by Okamoto et al. (2002) in cat serum and later detected in countries such as France and China, are a newly described virus species found at a high frequency in various animal species [5,9,12,43,44]. Zhu et al. (2011) reported that 12.5% (2/16) of serum samples tested positive for FeTTV, while Jarošová et al. (2015) later found a higher prevalence of 33.63% (37/110) in serum samples from Czechia [9,12]. A recent study conducted by Gao et al. (2023) demonstrated 36.67% (11/30) positivity rate in stool samples [43]. This study revealed an overall TTFeV-1 prevalence of 20.88% (19/91) in rectal swabs from domestic cats, with a slightly higher rate in kittens (23.21%) than in adult cats (17.14%). Phylogenetic analysis of our FeTTV sequences using the maximum likelihood method further revealed varying degrees of genetic similarity, with the sequences clustering into a large group of FeTTVs from domestic cats across China, France, the USA, and Czechia. Finally, sequencing results unveiled the genomic characteristics of six more *Etatorquevirus felid1*-associated strains, reflecting the genetic diversity of the virus in the region. We attempted to detect other felid torque teno virus species using predesigned primer sets, with samples previously isolated from chicken as a positive control; however, no positive results were found. These findings might serve as a foundation for future research into the rapid detection of FeTTV and the role of TTV in domestic cats.

A recent comprehensive study by Kraberger et al. (2021) pointed out the intricacy of Torque teno felid viruses’ evolutionary mechanisms in the *Felidae* family, with reference to recombination events stemming from simultaneous infections by *Anelloviridae* [45]. The co-occurrence of multiple anelloviruses and gyroviruses have been extensively documented across various studies, including those on feline [13] and human fecal samples [46], as well as rodent spleens [47]. Furthermore, Liu et al. (2022) revealed the coexistence of a bocaparvovirus-like virus and Anelloviridae members in the lymph nodes of the Amur leopard cat (*Prionailurus bengalensis euptilurus*), further confirming the significant genetic similarity of the bocaparvovirus to strains previously identified in diarrheic cats [48]. In a similar vein, substantial evidence has been presented so far indicating that various viruses, including Epstein–Barr virus, papillomavirus, and hepatitis C virus, may act as facilitating agents in the replication process of TTVs, potentially contributing to the development of diseases such as multiple sclerosis and carcinoma [8,49,50]. On the other hand, TTVs have been identified as components of the microbiome in healthy pregnant women, detected incidentally in sources like raw buffalo milk, and even associated with potential benefits for human health, such as reducing the risk of schizophrenia [51,52,53]. In this study, we documented the coexistence of DNA virus species from the *Parvoviridae* and *Anelloviridae* families and also showed the co-occurrence of anellovirus and gyrovirus species in diarrheic cats. However, our further statistical analysis demonstrated no statistically significant associations were observed between TTFeV1 and the other detected viruses. Taken together, we hypothesised that TTFeVs might be a substantial component of the felid gastrointestinal flora; however, interactions with some other pathogens could also fluctuate the viral load and potentially alter the virus’s role in feline diarrhea.

The first detection of the CAV-related strain (CAT-CAV, KC414026) in domestic cats was reported by Zhang and colleagues (2012), who detected recombination events within the partial C-terminus of the VP1 gene and UTR regions [10]. Then, a more recent complementary study investigated the occurrence of gyroviruses in domestic cats, identifying the presence of AvGyV2, GyV3, and GyV6 for the first time. Notably, the overall prevalence of GyVs was significantly higher in healthy cats (8.7%) than in diarrhoeic cats (3.8%) [13]. In this study, we observed an exceptionally high rate of Chicken Anemia Virus (35.16%) and AvGyV2 (73.63%) viral DNA positivity in cat stools. Furthermore, statistical analysis indicated the moderate level of association (*p* < 0.05) between chicken anemia virus and AvGyV2 in cat samples. A recent study by Yang et al. (2023) demonstrated the convergence of these two viruses in chickens, confirming that their co-application to SPF chickens exacerbates immunosuppression, pathogenicity, and viral replication of both viruses [54]. Taken together, we conjecture that a similar synergistic mechanism between these two gyroviruses could exist in other hosts, potentially leading to a higher prevalence of gyroviruses in domestic cats.

In this study, we successfully amplified a 351 bp nucleotide sequence, representing approximately 15% of the entire genome, which has provided valuable biological insights into the chicken anemia virus identified in the Turkish cat population. Phylogenetic analysis further demonstrated high variability among the sequences, with Fe-CAV33/TUR and Fe-CAV46/TUR clustering together in a single branch, whereas Fe-CAV43/TUR, Fe-CAV60/TUR, and Fe-CAV76/TUR formed a separate branch alongside a central Turkish cluster. A recent study by Song et al. (2024) analysed the genomic sequences of 28 CAV strains, identifying three primary groups and additional subgroups based on complete genome data [55]. Upon slight modification of this dataset and subsequent phylogenetic analysis based on partial nucleotide sequence, Groups I–III were distinctly identified; however, the bootstrap values were lower than anticipated (below 70), offering limited statistical support for the subdivision of Groups IIa–IIb and IIIa–IIIb. In conclusion, the primer set designed for this study effectively distinguishes groups; however, a longer amplicon may be required for the identification of subgroups.

The VP1 gene, which encodes the only structural protein of gyroviruses, plays an integral role in receptor binding and mediating interactions between the virus and its host [56,57]. Therefore, it is plausible that mutations in the VP1 protein could influence cell tropism and contribute to the emergence of immune escape mutants. An initial study by Renshaw et al. (1996) identified various point mutations by comparing cell culture-adapted CAV (isolate ConnB) with field isolates and detected a hypervariable region (HVR) between 139 and 151 that may alter the pathogenicity and transmissibility of viral strains [58]. Later, Todd et al. (2002) identified additional point mutations associated with viral pathogenicity in vivo at positions 75, 89, 125, 141, and 144, which may be related to the virus’s adaptation during interspecies transmission [59]. Although several strains have been previously reported in chickens in Türkiye [60,61], none have been identified in cats; thus, we conducted a comparative analysis of our strains alongside these reported, reference (Cuxhaven 1), and attenuated strains (clone 33, clone 34 and ConnB) based on the partial amino acid sequence of the VP1 protein. We identified several significant mutations. First, the V75I substitution was observed in Fe-CAV43/TUR, Fe-CAV60/TUR, and Fe-CAV76/TUR. Second, the I125L substitution was detected in Fe-CAV33/TUR and Fe-CAV46/TUR. Third, various alterations were found in hypervariable regions, particularly the K139Q and D144Q/E mutations. These mutations were frequently detected in other strains, including previously reported Turkish strains, and were less likely to be part of the feline host adaptation process. Therefore, we concluded that amino acid sequences clearly reflect the genetic diversity of CAV in Türkiye, highlighting the need for further genomic characterization to elucidate virus–host interactions.

AvGyV2 has been reported worldwide, and multiple studies have confirmed its presence in humans, dogs, cats, and ferrets, underscoring its high host range plasticity. [15,62,63,64,65,66]. Our phylogenetic analysis revealed significant diversity in the VP1 gene of Turkish strains, with FeAvGyV2-20/TUR grouping into clade 1 while the remaining strains clustered in clade 2, both consisting of isolates with no apparent geographical link. Furthermore, multiple sequence analyses based on the 203 deduced amino acid sequences revealed various mutations, which were interpreted in the context of significant alterations. Yao et al. (2017) drew insights into evolutionary trends of avian gyrovirus 2 in China by comparing the structural proteins of 17 strains and annotating hypervariable regions between positions 288 and 314, as well as a replication motif between positions 325 and 329 in VP1 protein [62]. Similarly, Liu et al. (2022) investigated the dynamics of host species transmission and identified several canine-related non-synonymous mutations [64]. We observed that our strains had several mutations in hypervariable regions, based on the reference sequence, which were common in other strains and the replication motif (FAAL) was well conserved. However, we detected V288I mutation in the same HV region, which was unique for FeAvGyV2-20/TUR. Taken together, it can be inferred that random mutations may be generated in this hypervariable region, which could contribute to interspecies transmission.

We compared our partial VP1 genome of GyV3 and GyV4 with GenBank sequences and found two main clades for each species due to limited data. All Turkish GyV3 strains were into Clade 1 with reference sequences, whereas two Chinese strains were outgrouped. Furthermore, Fe-GyV3-29/TUR and Fe-GyV3-90/TUR had the highest identity with a bird originated strain (99.53%, isolate BR_DF5), whereas Fe-GyV3-57/TUR and Fe-GyV3-75/TUR were closely related to domestic cat fecal samples [13,67]. This could be attributed to different sources of infection in each individual cat. On the other hand, the deduced amino acid sequence showed that all four strains were identical to the partial VP1 protein of the reference genome, indicating that the mutations in the genome remain synonymous. The infectivity of GyV3 and its broad host-range capacity have been extensively studied in chickens and mice, showing that GyV3 targets hematopoietic cells in the bone marrow, leading to severe aplastic anemia and immunosuppression, as well as hepatitis and gastroenteritis in both hosts [68,69]. Furthermore, mutations in the process of infection in mice revealed a hypervariable region in the C- terminus of VP1, which could not be detected in this study [68]. Taken together, our findings suggested that cats are naturally infected with GyV3 from multiple sources at a low prevalence, without any evidence of adaptation. Given that GyV3 has been detected in human faeces [46], its presence is particularly noteworthy due to the close interactions between humans and animals, which may facilitate viral transmission. The identification of gyrovirus DNA in fecal samples from diarrheic cats emphasizes the importance of continued monitoring to assess potential health risks.

Partial VP1 sequences were used to generate a phylogenetic tree, where Turkish GyV4 sequences grouped with the reference strain in Clade 1 and showed the closest relationship to a strain previously isolated from chicken meat in Brazil [70]. Two strains, isolated from ferrets and chickens, exhibited high identity and formed Clade 2. Then, multiple sequence alignments were conducted based on the 226 bp amino acid sequence of the VP1 gene, which is relatively closer to the N-terminal end. Our analysis revealed a unique Y21H mutation in Fe-GyV4-37/TUR, while the other two sequences exhibited common alterations. Due to limited data, we could not identify any associations between strains based on either geographical location or host species. Notably, we report the occurrence of GyV4 in cat samples for the first time.

This study has several limitations. Faecal samples were obtained from the Faculty Animal Hospital and six private veterinary clinics across different districts of Sivas, Türkiye. Most specimens originated from cats exhibiting mild to severe diarrhea; however, a standardized clinical scoring system was not applied by participating practitioners. The majority of samples were collected from rural clinics, and owners of cats that recovered typically did not return for follow-up examinations despite our recommendations. As the sampling was restricted to diarrheic animals, the prevalence of anelloviruses and gyroviruses among healthy cats could not be evaluated. Moreover, sequence and phylogenetic analyses were conducted using partial VP1 fragments, which limited the ability to perform comprehensive structural comparisons among all gyrovirus species and required separate analyses for each viral group.

## 5. Conclusions

This investigation provides new insights into the prevalence and molecular characteristics of anelloviruses and gyroviruses in diarrheic domestic cats. The detection of TTFeV1, CAV, AvGyV2, GyV3, and GyV4 broadens the recognized diversity of circular DNA viruses associated with feline disease. These findings highlight the necessity of continued molecular surveillance and broader epidemiological studies, including healthy populations, to clarify the pathogenic and ecological roles of these viruses and to support both animal and public health.

## Figures and Tables

**Table 1:** PCR primers and annealing temperatures used for the detection of feline panleukopenia virus, feline anelloviruses, and gyroviruses in fecal samples from diarrheic cats.

Primer Name	Sequence (5′–3′)	Target Gene	Amplicon Size (bp)	Annealing Temp. (°C)
**TTFeV1-81F** **TTFeV1-410R**	TAGTCATGGAACTAGGAGCGC CTGAAATGTTGGGTGTAGTCTC	TATA Box-*ORF2 gene*	330 bp (This study)	55
**TTFeV2-837F** **TTFeV2-1451R**	ATACCACCACCATCTAGCACAC CTTTTTATGAGCGGTTGGGGAG	*ORF1-ORF2 genes*	615 bp (This study)	57
**CAV 974F** **CAV 1328R**	GTAGACGAGCTTTTAGGAAG GAGGGCAYGTTATTATCTAG	*VP1 gene*	355 bp (This study)	50
**AvGy2-1360F** **AvGy2-1969R**	CTTGCAGGGGTGCCAATGGT GCTAGGAAATGACCAGGGTGC	*VP1 gene*	610 bp (This study)	51
**Gy3-1101Fn** **Gy3-1744Rn**	ACCCCTATAACGCGATTAACCT TGGTATTGTGGTTTCATTAGCTGG	*VP1 gene*	644 bp (This study)	53
**Gy4-1259Fn** **Gy4-1938Rn**	CTGAAACTTCTGCTTTTAGGGT CGTTTCACTCAATCCAGTAGCT	*VP1 gene*	330 bp (This study)	52
**Hfor** **Hrev**	CAGGTGATGAATTTGCTACA CATTTGGATAAACTGGTGGT	*VP2 gene*	630 bp [22]	49

**Table 2:** PCR detection of viruses in adults and kittens, presented as number of positive samples and percentage of the group, with overall percentages for all samples.

Virus	Adults (*n* = 35)	Kittens (*n* = 56)	Total (*n* = 91)
FPV	7/35 (20.00%)	30/56 (53.57%)	37/91 (40.65%)
TTFeV1	6/35 (17.14%)	13/56 (23.21%)	19/91 (20.88%)
CAV	11/35 (31.43%)	21/56 (37.50%)	32/91 (35.16%)
AvGyV2	25/35 (71.43%)	42/56 (75.00%)	67/91 (73.63%)
GyV3	1/35 (3.86%)	3/56 (5.36%)	4/91 (4.40%)
GyV4	1/35 (3.86%)	2/56 (3.57%)	3/91 (3.30%)

## Data Availability

The obtained partial genome sequences of anelloviruses in this study are available under the accession numbers PQ281752–PQ281774 in GenBank.
